# Influence of early goal-directed therapy using arterial waveform analysis on major complications after high-risk abdominal surgery: study protocol for a multicenter randomized controlled superiority trial

**DOI:** 10.1186/1745-6215-15-360

**Published:** 2014-09-16

**Authors:** Leonard Montenij, Eric de Waal, Michael Frank, Paul van Beest, Ardine de Wit, Cas Kruitwagen, Wolfgang Buhre, Thomas Scheeren

**Affiliations:** University Medical Center Utrecht, Heidelberglaan 100, 3584 CX Utrecht, The Netherlands; Albert Schweitzer Hospital, Albert Schweitzerplaats 25, 3318 AT Dordrecht, The Netherlands; University Medical Center Groningen, Hanzeplein 1, 9700 RB Groningen, The Netherlands; Maastricht University Medical Center, P. Debyelaan 25, 6229 HX Maastricht, The Netherlands

**Keywords:** High-risk surgery, Early goal-directed therapy, Hemodynamic monitoring, Hemodynamic optimization, Tissue oxygen delivery, Randomized controlled trial, Outcome, Quality of life, Cost-effectiveness

## Abstract

**Background:**

Early goal-directed therapy refers to the use of predefined hemodynamic goals to optimize tissue oxygen delivery in critically ill patients. Its application in high-risk abdominal surgery is, however, hindered by safety concerns and practical limitations of perioperative hemodynamic monitoring. Arterial waveform analysis provides an easy, minimally invasive alternative to conventional monitoring techniques, and could be valuable in early goal-directed strategies. We therefore investigate the effects of early goal-directed therapy using arterial waveform analysis on complications, quality of life and healthcare costs after high-risk abdominal surgery.

**Methods/Design:**

In this multicenter, randomized, controlled superiority trial, 542 patients scheduled for elective, high-risk abdominal surgery will be included. Patients are allocated to standard care (control group) or early goal-directed therapy (intervention group) using a randomization procedure stratified by center and type of surgery. In the control group, standard perioperative hemodynamic monitoring is applied. In the intervention group, early goal-directed therapy is added to standard care, based on continuous monitoring of cardiac output with arterial waveform analysis. A treatment algorithm is used as guidance for fluid and inotropic therapy to maintain cardiac output above a preset, age-dependent target value. The primary outcome measure is a combined endpoint of major complications in the first 30 days after the operation, including mortality. Secondary endpoints are length of stay in the hospital, length of stay in the intensive care or post-anesthesia care unit, the number of minor complications, quality of life, cost-effectiveness and one-year mortality and morbidity.

**Discussion:**

Before the start of the study, hemodynamic optimization by early goal-directed therapy with arterial waveform analysis had only been investigated in small, single-center studies, including minor complications as primary endpoint. Moreover, these studies did not include quality of life, healthcare costs, and long-term outcome in their analysis. As a result, the definitive role of arterial waveform analysis in the perioperative hemodynamic assessment and care for high-risk surgical patients is unknown, which gave rise to the present trial. Patient inclusion started in May 2012 and is expected to end in 2016.

**Trial registration:**

This trial was registered in the Dutch Trial Register (registration number NTR3380) on 3 April 2012.

**Electronic supplementary material:**

The online version of this article (doi:10.1186/1745-6215-15-360) contains supplementary material, which is available to authorized users.

## Background

### High-risk surgery

Both the extent of the surgical procedure and the presence of comorbidities increase the risk of an adverse outcome after surgery. Moreover, approximately 12.5% of all surgical patients are considered as ‘high-risk’ [1, 2]. The majority of perioperative complications and death occur in this minority of patients [1]. With increasing age and comorbidities in the general population, the number of high-risk surgical patients is expected to grow. Approximately 50% of these surgical patients develops one or more perioperative complications, and mortality in this specific patient group is around 5% [1–6]. Major complications such as myocardial infarction, cardiac failure, stroke, pneumonia, renal failure, or sepsis, involve a significant burden to the patient and to hospital facilities during the hospitalization period [3]. After discharge, these complications may result in long-term disability, cognitive impairment and even death, significantly reducing quality of life and increasing healthcare consumption [3].

### Hemodynamic monitoring in high-risk surgery

Surgical patients are subject to the effects of anesthesia, direct surgical trauma, inflammation, fluid shifts, and blood loss. In extensive surgical procedures these effects are enhanced, which may impair oxygen delivery to organs and tissues. Patients with significant comorbidities have limited cardiopulmonary reserve and are therefore more susceptible to the consequences of surgical stress [7]. An imbalance in tissue oxygen delivery and demand may occur, which promotes the incidence of complications [8–10]. This emphasizes the need for appropriate hemodynamic monitoring and optimization therapy to maintain adequate tissue oxygen delivery in perioperative care for this high-risk group.

Cardiac output (CO) is the principal determinant of tissue oxygen delivery. Continuous monitoring of CO would therefore be valuable for each high-risk surgical patient. A number of CO measurement techniques are available. Pulmonary artery thermodilution remained the standard technique for a number of decades [11, 12]. This technique is, however, invasive and associated with potentially life-threatening complications which limits its use in clinical practice [11, 12]. Transesophageal echocardiography (TEE) can be used to measure CO, but is also rather invasive, does not provide CO continuously, and requires specific skills that are not included in the training of each anesthesiologist or intensive care physician [13]. Therefore, TEE is predominantly used in cardiac surgery. Esophageal Doppler probes for continuous CO measurement are easier to use but not readily tolerated by conscious patients. Other minimally invasive CO techniques such as bioimpedance are not yet sufficiently reliable for use in clinical practice [13].

Arterial waveform analysis (AWA) provides a less invasive and more practical alternative to conventional techniques of CO monitoring. CO is derived from the arterial pressure waveform and only requires an arterial line, which is almost routinely placed in high-risk surgical patients in the operating room (OR) and intensive care unit (ICU) [13, 14]. Moreover, the AWA technique enables dynamic preload assessment in mechanically ventilated patients. Assessment of cardiac preload or fluid responsiveness is the first step in optimizing CO [14, 15]. Both AWA-based CO measurement and dynamic preload assessment have been validated in a variety of clinical settings [14, 16]. The technique is therefore potentially valuable for advanced hemodynamic monitoring and fluid optimization of high-risk, non-cardiac surgical patients [17].

### Early goal-directed therapy

Adequate strategies to correct and improve hemodynamic variables should be available, since monitoring without therapeutic consequences is not enough. Early goal-directed therapy (EGDT) refers to the preemptive use of predefined target values for hemodynamic optimization in order to maintain tissue oxygen delivery. A target variable is continuously monitored and immediately corrected using a treatment algorithm if the value decreases below a predefined threshold. Especially CO or CO-derived variables have been used for this purpose. The treatment algorithms used in EGDT guide fluid therapy and inotropic support, in order to improve the patient’s hemodynamic status. With the emergence of AWA techniques, a new, promising CO measurement technique became available for EGDT [14, 18].

### Previous literature

Before the start of the study we performed a systematic review of the literature to evaluate all available evidence on the use of EGDT in elective, high-risk, abdominal surgery (Additional file 1). The results indicated that EGDT is a promising strategy to reduce morbidity and even mortality in high-risk surgery. There are, however, several limitations to the previous literature which may explain why EGDT has never been widely implemented into routine practice [19]. First, the results from most studies are outdated and not applicable to the current standard of care. In studies from 1988 to 2000, morbidity and mortality are much higher in comparison with current practice, even in the intervention groups. The same applies for length of stay in the hospital. Second, pulmonary artery catheterization has been used for the purposes of EGDT at that time. As described before, this technique is limited to specific types of surgery and not broadly accepted for perioperative monitoring in non-cardiac surgery [11–13, 20]. Two studies applying arterial waveform analysis for EGDT purposes were small and performed in a single center [4, 6]. Moreover, minor complications, such as short-lasting intraoperative hypotension or urinary tract infection, are included as an outcome measure. These complications are not associated with increased long-term morbidity and mortality and should be evaluated separately. One final but major limitation applies to all of the studies described. They lack information about the effect of EGDT on long-term outcome, quality of life, and utilization of healthcare resources. This hinders a cost-effectiveness analysis. EGDT requires the purchase of CO monitoring equipment which consists of specific patient monitors and arterial blood pressure sensors. Additional costs may be the result of an increased use of inotropic support or other therapeutic consequences of EGDT.

Since the start of the trial in May 2012, a number of interesting studies have been published which partially addressed the shortcomings mentioned above [5, 21–23]. Two studies demonstrated that the implementation of a goal-directed strategy was cost-effective [21, 22]. In addition, two multicenter studies were performed in which a reduction in complications was shown [5, 23]. However, minor complications were included, and the number of patients was rather small in these studies.

### Added value of the proposed study

The present study is a multicenter, randomized controlled trial in a relatively large group of patients and performed in both university and non-academic teaching hospitals. Patients scheduled for elective high-risk abdominal surgery are included. They represent an extensive group of patients at risk for adverse postoperative outcomes. The primary aim is to reduce the number of major complications that are associated with an increased short-term and long-term morbidity and mortality. These complications are expected to comprise a major burden to quality of life, healthcare costs, and long-term outcome, which are included as secondary outcome parameters. AWA-based CO monitoring techniques are used to assess tissue oxygen delivery, which are minimally invasive and easy-to-use. The treatment algorithm is straightforward and suitable for each anesthesiologist, ICU physician, and anesthesia or ICU nurse. The results of this study may therefore be useful in guiding evidence-based implementation of EGDT.

### Objective

The primary objective of the study is to investigate the effect of perioperative EGDT using arterial waveform analysis on a composite endpoint of major complications, including mortality after high-ris, abdominal surgery, in comparison with standard care. Secondary endpoints are minor complications, length of stay in the hospital, length of stay in the ICU or post-anesthesia care unit (PACU), long-term outcome, quality of life (QOL), and cost-effectiveness. We hypothesize that perioperative EGDT using AWA reduces the number of major complications after high-risk abdominal surgery in comparison with standard care.

## Methods/Design

### Trial design

The EGDT trial is designed as a multicenter, randomized, controlled superiority trial with two parallel groups in a 1:1 allocation. The trial is unblinded for the clinicians involved, the patients, and the observers.

### Study setting

Currently, the study is performed in three hospitals in The Netherlands: Albert Schweitzer Hospital (Dordrecht, The Netherlands), University Medical Center Groningen (Groningen, The Netherlands), and University Medical Center Utrecht (Utrecht, The Netherlands). The Maastricht University Medical Center (Maastricht, The Netherlands) is preparing study participation. Additional participating centers may be added in the future.

### Eligibility criteria

Eligible are patients scheduled for elective high-risk abdominal surgery. Two groups of patients are considered high-risk: patients undergoing extensive procedures comprising a high risk for an adverse postoperative outcome, irrespective of the condition of the patient (group 1); patients undergoing moderately extensive procedures that suffer from significant comorbidity (group 2). These procedures comprise a moderately high-risk for an adverse postoperative outcome in healthy patients, but are considered high-risk for patients with significant comorbidity.

Inclusion criteria are:

1. Group 1: patients scheduled for the following operations in which postoperative observation in the ICU/PACU is needed, irrespective of their American Society of Anesthesiologists (ASA, Table 1) physical status: esophagectomy, pancreaticoduodenectomy, open abdominal aorta aneurysm repair, and major abdominal resections for soft tissue malignancy.
2. Group 2: patients with ASA physical status III or IV scheduled for the following surgical procedures in which postoperative observation in the ICU/PACU is needed: gastrectomy, colorectal resections for carcinoma, and other extensive upper or lower abdominal surgery (such as ileocystoplasty or debulking surgery for ovarian cancer).

**Table 1:** **American Society of Anesthesiologists (ASA) physical status**

ASA physical status	Description
I	Normal, healthy patient
II	Patient with mild systemic disease which does not affect daily functioning (such as controlled hypertension, stable chronic obstructive pulmonary disease (COPD), well-regulated diabetes mellitus, mild obesity)
III	Severe systemic disease which affects daily functioning (such as coronary artery disease, heart failure or COPD limiting exercise tolerance, morbid obesity)
IV	Systemic disease that is a constant threat to life (such as invalidating COPD or heart failure, unstable angina pectoris)
V	Moribund patient

Patients will be excluded if they meet one of the following criteria: patients with aortic insufficiency > grade 1, patients who are under 18 years-old, patients with cardiac arrhythmias (such as atrial fibrillation or flutter, or ventricular tachycardia), patients requiring emergency surgery, patients in which cardiac output measurement is mandatory for therapeutic decisions, or contraindication for passive leg raising in the entire postoperative period.

### Randomization

A randomization procedure is used to allocate patients to standard care (control group) or standard care with EGDT (intervention) in a 1:1 ratio. The randomization is stratified according to hospital and type of surgery. In each stratum, a block randomization with variable block size is applied using a computer.

### Standard care

Patients assigned to standard care will be treated according to local routine, with the following conditions: 1) after induction of anesthesia and tracheal intubation, patients are mechanically ventilated (volume control) with a fixed tidal volume of 8 ml/kg^-1^ (ideal body weight, IBW) throughout the procedure, adjusting respiratory rate to maintain normocapnia; 2) an arterial line, central venous line, and urine catheter are placed, 3) hypotension after induction is treated preferably with crystalloids, eventually with colloids, with a maximum of 500 ml. Ongoing hypotension is treated with a continuous infusion of norepinephrine. Sudden, short-lasting hypotension is treated with phenylephrine or ephedrine depending on local routine and indication; and 4) transfusions with erythrocytes will be applied according to the 4-5-6 rule (Additional file 2).

Therapy is aimed at maintaining: S_p_O_2_ ≥ 95%, mean arterial blood pressure (MAP) ≥ 60 mmHg or ≤ 25% below baseline in cases of preexisting hypertension, and heart rate (HR) < 100/min or ≤ 25% above baseline.

The use of epidural analgesia, enhanced recovery after surgery (ERAS) protocols, corrections for preoperative fasting, and basic fluid regimes is allowed but not obligatory. Serum lactate, central venous oxygenation saturation, and other laboratory parameters may be used to the discretion of the attending anesthesiologist or ICU physician.

### Intervention

Patients assigned to EGDT are treated according to the same conditions as defined for the standard group. An EGDT algorithm is added to standard care in order to maintain tissue oxygen delivery in the perioperative period. After induction of anesthesia in the OR, an AWA technique is applied for continuous CO measurement and dynamic preload assessment. The choice of AWA technique depends on the institution in which the study is performed. CO values are indexed to the patients’ body surface area. For each patient, an age-dependent target cardiac index (CI) is determined with the following criteria: age < 60 years, target CI ≥ 2.8 l/min/m; age between 60 and 75 years, target CI ≥ 2.6 l/min/m; or age > 75 years, target CI ≥ 2.4 l/min/m.

**Figure 1:**
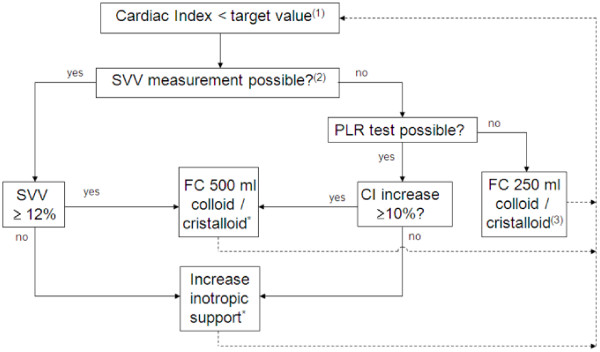
**EGDT algorithm.** ^(1)^target value depends on age: age < 60 years: target CI ≥ 2.8 l/min/m^2^; age between 60 and 75 years: target CI ≥ 2.6 l/min/m^2^; or age > 75 years: target CI ≥ 2.4 l/min/m^2^. ^(2)^”no” in the presence of spontaneous breathing activity, tidal volume < 8 ml/kg, or breathing frequency > 16/min. ^(3)^CI increases: repeat FC. If not: increase inotropic support. CI = Cardiac Index, FC = Fluid Challenge, PLR = Passive Leg Raising, SVV = Stroke Volume Variation, Tidal volumes are in ml/kg ideal body weight (IBW); IBW is calculated as 22 × L^2^ (L = length in meters).

If the CI drops below the target value, a treatment algorithm is used in order to restore the CI above the threshold (Figure 1). In this algorithm, the dynamic preload parameter stroke volume variation (SVV) is used to determine whether the patient should receive a fluid challenge (FC), or whether inotropic support should be started or increased. Continuous infusion of norepinephrine and/or dobutamine should be used as inotropic support. SVV cannot be used in the following circumstances: spontaneous breathing activity, tidal volume <8 ml/kg, and/or breathing frequency >16/min^-^.

**Figure 2:**
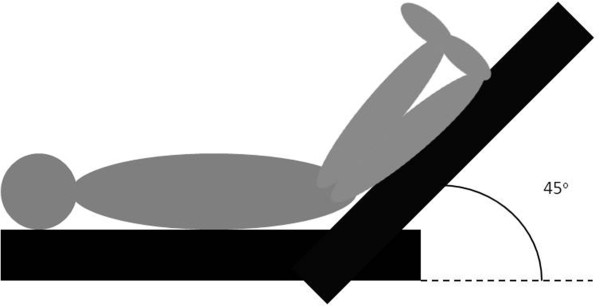
**Passive leg raising (PLR) test.** The patient is transferred to the PLR position, in which the legs are lifted at an angle of 45° for 120 seconds. This should be performed by pivoting the entire bed.

In these circumstances, a passive leg raising (PLR) test is used to guide fluid or inotropic support [24–26]. The procedure is explained in Figure 2. If the CI increases 10% or more during PLR, a 500 ml FC is given. If not, inotropic support is started or increased. If both measurement of SVV and PLR testing are not possible, a small 250 ml FC is given. If the CI subsequently increases, another 250 ml FC is given. If the CI does not increase, inotropic support is started or increased. Treatment according to this EGDT algorithm starts after induction of anesthesia in the OR and is maintained until discharge from the ICU/PACU, with a maximum of 24 hours from induction of anesthesia.

### Modifications

In case of any newly developed arrhythmia in patients in the intervention group, CO and SVV measurements obtained with AWA are unreliable. Therefore, treatment according to the EGDT algorithm stops as long as the arrhythmia persists. If a patient in the intervention group develops unwanted tachycardia, arrhythmia, or (suspected) myocardial ischemia due to inotropic support applied within the EGDT algorithm, inotropic support needs to be reduced or stopped, irrespective of the CI. In addition, a FC should be withheld or stopped if pulmonary edema or cardiac decompensation is suspected, irrespective of the CI.

### Protocol adherence

The EGDT algorithm is easy to use and rather straightforward, yet extensive training of nurses and physicians involved in the care of patients allocated to EGDT will be performed before the start of the study in each participating center. Moreover, a number of nurses and physicians from each institution have been involved in the trial from the beginning and are thoroughly informed about the procedures. These ‘trial experts’ can be consulted at any time and will monitor the appropriate application of the EGDT algorithm.

### Outcome measures

The primary outcome measure is the number of major complications per patient within the first 30 days after surgery, including mortality. An overview of all major complications can be found in Table 2. The complications within this composite endpoint are associated with increased long-term morbidity and mortality [3].

**Table 2:** **Major complications within the first 30 days after surgery embedded in the composite primary endpoint**

Category	Complication	Criteria
Mortality	30-day mortality	Mortality within the first 30 days after surgery
Cardiovascular	Cardiac arrest	Cardiac arrest, electromechanical dissociation, ventricular fibrillation, or ventricular tachycardia without output
	Acute myocardial infarction	According to the criteria for acute myocardial infarction (see Universal Definition of Myocardial Infarction [27])
	Acute pulmonary edema	Diagnosis confirmed by X-ray
	Stroke	Focal brain injury that persists for >24 hours, combined with an increase in disability of at least one grade on the modified Rankin scale [28], confirmed by cerebral CT-scan
Respiratory	Prolonged mechanical ventilation	Postoperative mechanical ventilation for more than 24 hours (any cause), including non-invasive pressure support ventilation through a face-mask
	Pulmonary embolism	Confirmed by CT-scan
	Pneumonia	Confirmed by X-ray and treated with antibiotics
	Respiratory failure	Any other respiratory complication requiring mechanical ventilation (including non-invasive pressure support ventilation)
Renal	Acute kidney injury	Stage 2 or 3 acute kidney injury, according to the Acute Kidney Injury Network [29]
Gastrointestinal	Anastomotic leak	Diagnose made and confirmed by surgeon
	Other	Any other complication requiring additional surgery
Infection	Wound	Diagnosis made and confirmed by surgeon
	Severe sepsis	See International Sepsis Definitions Conference [30]

The following secondary outcome measures are considered: the grade of major complications occurring in the first 30 days after surgery according to the Accordion Severity Grading system [31]; the number of the following minor complications within the first 30 days after surgery: arrhythmia (requiring intervention), deep venous thrombosis (confirmed by ultrasonography), urinary tract infection (confirmed by urinalysis and treated with antibiotics), prolonged ileus (diagnose made and confirmed by surgeon), herniation or other prolonged wound healing (diagnosis made and confirmed by surgeon); the total amount of fluid administered in the first 24 hours after surgery (crystalloids, colloids, and blood products); inotropic support in the first 24 hours after surgery (amount and duration of infusion); hemodynamic (blood pressure, HR, CI, SVV, mean urine output) and laboratory (central venous oxygen saturation and pCO_2_, serum lactate, troponin) variables in the first 24 hours after surgery; length of stay in the hospital and in the ICU/PACU; continuation of care at intermediate care units (level of care in between the ward and ICU/PACU); number and length of ICU/PACU or intermediate care readmissions; QOL; cost-effectiveness; long-term outcome: 3, 6, and 12 months complications and mortality.

### Data collection

Before the operation demographic data, ASA physical status, medical history, and drug usage are collected from the patient’s medical records.

A number of hemodynamic variables (blood pressure, HR, CI, and SVV if applicable) are recorded each hour during the first 24 hours after induction. In the EGDT group, these variables are also recorded before and after interventions to correct a drop in CI below the target value. Fluid balance in the first 24 hours is evaluated by recording the amount of fluids infused (crystalloid, colloid, and blood products) as well as urine output and blood loss. In addition, the use of inotropes is registered (type, amount, and duration of infusion). The following laboratory variables are determined after induction, during surgical closure, after admittance to the ICU/PACU, and the morning after surgery: serum hemoglobin, lactate, and full blood gas analysis (arterial and venous). After admittance to the ICU/PACU, the following additional blood values are determined in order to calculate the Sequential Organ Failure Assessment (SOFA) score [32]: serum creatinine, bilirubin, and platelet count. The morning after surgery, serum troponin and creatinine levels are measured. The Therapeutic Intervention Scoring System (TISS) is calculated after admittance to the ICU/PACU [33]. Variables are derived from the patient monitoring systems used in the operating room and ICU/PACU, or from the OR and ICU/PACU record systems.

The following information is obtained in the first 30 days after surgery: all-cause mortality, major and minor complications, if applicable; duration of postoperative mechanical ventilation (hours); length of stay in the in the hospital (days) and ICU/PACU (hours); length of stay in the in the hospital (days) and ICU/PACU (hours) according to fit-for-discharge criteria [5] (Additional file 3); continuation of care at intermediate care units (hours); number of readmissions to the ICU/PACU or intermediate care unit.

All information can be obtained from the patients’ medical records. Patient follow-up with respect to complications and length of stay is active.

QOL will be assessed with the EQ-5D and Short Form (SF) 36 questionnaires before surgery and 1, 3, 6, and 12 months after surgery. Depending on the patient’s preference, the questionnaires are sent to the patient by email or conventional mail, or the patient is called by an interviewer. Patients who do not respond will receive a maximum of two phone calls as a reminder. Before contacting or sending the questionnaires to the patient, confirmation will be obtained that the patient is still alive.

Both healthcare costs (treatment costs including equipment, hospital stay, and re-interventions) and non-healthcare costs (costs as a result of absence from work) will be analyzed. Additional equipment and staff costs associated with EGDT will be monitored to assess incremental costs associated with EGDT. Direct healthcare costs will be calculated by multiplying the volume of (healthcare) resources use with its cost, using standard reference prices for economic evaluation in healthcare whenever available. Information on the following direct costs will be collected from the medical records: blood sample measurements, cultures and other investigations (such as chest films, CT-scans, echography, and ECG), interventions (such as reoperation, catheterization, chest drainage, and antibiotic treatment), and discharge destination (home, nursing home, rehabilitation). Data on absenteeism from work will be collected using parts of the Short-Form Health and Labor Questionnaire (SF-HLQ). The following three questions are added to the SF-HLQ: how many times did you visit your general practitioner? How many times did you visit a doctor in the hospital? Have you been admitted to the hospital? These questions are included in the QOL questionnaire described above. Indirect costs associated with production losses will be estimated using the friction cost method.

Long-term outcome is determined with a letter to the general practitioner of the patient after 3, 6, and 12 months. In this way, mortality and morbidity in the period between 30 days and one year after the operation is determined.

### Statistical analysis and sample size calculation

A study of the hospital data of one of the participating centers revealed that the number of major complications embedded in the composite primary endpoint was 0.58 per patient, and that a Poisson distribution was applicable. We aim to reduce the number of major complications per patient by 30% (from 0.58 to 0.41). Using Poisson regression with an assumed two-sided error of 5%, we calculated that a sample size of 226 patients in each group will be needed to detect this reduction with a power of 80%. For the multicenter character of the study, a 20% correction was made, since the variance between the participating centers is unknown. This results in a total sample size of 542, or 271 patients in each group.

Statistical analysis will be performed using SPSS version 21.0 for Windows (SPSS Inc, Chicago, Illinois, United States). The first analysis will be performed by combining the results from the participating centers. After this, the results of the individual centers are analyzed to detect possible differences. The primary analysis consists of a comparison of the primary endpoint (number of complications per patient) between the control group and the EGDT group using Poisson regression. According to secondary endpoints, data will be checked for normal distribution and presented as mean +/-standard deviation when normally distributed, and as median +/-interquartile range when not normally distributed. Relative risks are presented with 95% confidence intervals. Categorical data are tested with Fisher’s exact test. Continuous data are tested with the independent samples t-test when normally distributed, and with the Mann-Whitney U test if not normally distributed. A *P* value <0.05 is considered statistically significant. Analysis will be carried out according to the intention-to-treat principle.

For the quality-of-life assessment, the mean EQ-5D score and the individual SF-36 subscale scores are determined, and a comparison will be made between the standard group and the EGDT group. In addition, mean change scores are determined and evaluated over time. For the economic evaluation, especially the EQ-5D, is of importance as utilities and consequently, quality-adjusted life years (QALY), can be elicited using this generic quality-of-life questionnaire. The incremental cost-effectiveness ratio (ICER) will be expressed as cost differences between groups divided by differences in numbers of serious complications between groups (cost per complication averted). The incremental cost-utility ratio (ICUR) will be expressed as cost differences between groups divided by differences in QALY’s gained between groups (cost per QALY). Confidence intervals will be determined using bootstrapping. A cost-effectiveness acceptability curve will be drawn using this bootstrap sample.

### Institutional Review Board approval and informed consent

The trial has been reviewed and approved by the institutional review board (IRB) of the University Medical Center Utrecht (The Netherlands). Furthermore, local IRB approval has been obtained in the participating centers for the local applicability of the protocol. Details regarding the IRB approval are presented in Additional file 4. Written informed consent will be obtained from all patients.

### Risk assessments

The treatment algorithm introduced in the patients assigned to EGDT includes measurement of cardiac output, SVV, and PLR as a test. These measurements are performed using the arterial catheter, which is routinely placed in patients undergoing high-risk, non-cardiac surgery. Therefore, no additional catheters are placed in comparison with routine hemodynamic monitoring. EGDT involves fluid therapy and inotropic support, which are commonly used in patients undergoing high-risk surgery. Additional risk associated with its use in the treatment algorithm is therefore not likely in comparison with routine practice. The systematic review of the literature performed before this study (Additional file 1) reported adverse events due to the use of pulmonary artery catheters, which are not used in this study. Only one study reported myocardial ischemia due to use of inotropic support, which disappeared after stopping inotropic infusion [6]. In the other studies however, the results point to a lower incidence of myocardial ischemia, although significant reductions have not been reported. Adverse events with respect to fluid overload have not been described in the systematic review. Despite this, precautions have been taken in the EGDT algorithm to prevent the potentially harmful effects of excessive fluid and inotropic support.

### Safety procedure

Events that meet the criteria for ‘severe adverse events’ (SAE’s) are monitored and reported to the accredited IRB that approved the protocol and to a Data Safety Monitoring Board (DSMB). The major complications embedded in the primary outcome measure meet these criteria and are considered ‘expected SAE’s’. Cumulative data on these expected SAE’s are reported biannually. Mortality and all other ‘unexpected SAE’s’ are reported immediately. The DSMB has been created to assess the safety of the interventions and to monitor the overall conduct of the study. In addition, the DSMB assists and advises the investigators in protecting the validity and credibility of the trial. The members of the DSMB for this trial are an epidemiologist and biostatistician, an anesthesiologist and intensivist, and a gastrointestinal surgeon. The DSMB members will meet every six months in closed and open sessions. After each meeting the DSMB may decide the following: the trial is allowed to continue without further action, the trial is allowed to continue with recommendations which will be evaluated in the next meeting, or recruitment of patients is stopped since accumulating data points to an adverse effect in the intervention arm. The study will not stop if accumulating data points to a beneficial effect in the intervention arm. The roles and responsibilities of the DSMB are described in detail in a DSMB charter.

## Discussion

### Trial context

There is an urgent need for a multicenter, randomized controlled trial, evaluating the application of AWA-based hemodynamic monitoring in goal-directed strategies for high-risk surgical patients [14, 34, 35]. Ideally, the algorithm used for EGDT should be user-friendly and easily applicable in order to enhance the implementation in daily care. Moreover, this trial should include a large group of patients in order to have sufficient power to demonstrate differences in clinically relevant outcome parameters. Finally, long-term effects in terms of QOL and healthcare costs should be considered. So far, this ‘ideal’ trial has not been performed. The presented study protocol attempts to include the important features mentioned, however, a number of aspects of the trial design however need to be discussed in detail. First we try to elucidate the clinical choices made in the selection of patients, the EGDT algorithm, care in the control group, and safety. Next, we will discuss a number of methodological issues with respect to the study design, primary outcome measure, blinding, and discharge criteria.

### Clinical considerations

#### Patient population

Each year a large number of patients are scheduled for extensive abdominal surgery in a variety of hospitals. The postoperative problems seen in the postoperative period involve a major burden to patients and healthcare costs, and are acknowledged by many clinicians in the field of anesthesia, critical care, and surgery. Yet there is ongoing debate if and which type of advanced hemodynamic monitoring should be applied in these patients. In contrast, transesophageal echocardiography and pulmonary artery catheterization are extensively used in patients undergoing cardiac surgery, while the risk for postoperative complications or mortality is often lower. Therefore, we chose major abdominal surgery as the target population in this study. Hepatic resections are considered high-risk abdominal surgery, especially due to bleeding complications. Intraoperatively, however, the use of fluids is highly restrictive and the algorithm described in this study is not suitable for application in this specific patient category. Therefore, we decided to exclude patients scheduled for hepatic resection.

#### EGDT algorithm

The algorithm was designed to be as easy as possible, to enhance the applicability in the various types of hospitals performing high-risk abdominal surgery. Basically, the algorithm includes three steps: 1) detect a drop in CI below the target value, 2) determine the intervention to restore the CI, and 3) evaluate if the CI increases above the target value.

The AWA technique used is not prescribed, but depends on the preference of the hospital. This will enhance the willingness to implement EGDT in clinical practice, since many hospitals already have AWA monitoring systems for other clinical purposes. In addition, EGDT is the intervention under investigation, not a specific device. AWA algorithms continue to improve and new devices will emerge in the coming years [36, 37].

We decided to use CO, indexed to Body Surface Area (BSA(m^2^) = 0.20247 × Height(m)^0.725^ × Weight(kg)^0.425^), as the target variable in the EGDT algorithm. CO gradually decreases with increasing age [38, 39]. Since age varies considerably in the target population, we decided to use different target CI values in three age categories. Assessment of fluid responsiveness is the first step in optimizing CO. An SVV measurement can be used for this purpose, if a number of criteria for mechanical ventilation are met [14, 15]. In the OR, this will be the case in most situations. In the ICU or PACU however, patients are weaned from the ventilator, which hinders the reliability of dynamic preload assessment. In these circumstances, PLR testing provides a suitable alternative, if properly performed [24–26]. If the SVV criteria are not met and PLR testing is not possible, we decided to use a small FC to prevent unwanted volume loading in unresponsive patients. This small FC can be repeated if the patient responds well. Otherwise, inotropic support is used to restore the CI. Overall, SVV and PLR testing are important in the algorithm, which emphasizes the role of cardiac preload in treating low CO. Preload optimization is, however, not the primary target in this trial, since CO represents the most direct way to determine tissue oxygen delivery in the perioperative period [13].

Central venous oxygen saturation (S_cv_O_2_) has been shown to be effective as a target variable in a number of goal-directed strategies in critical care, and has been considered as target variable for this trial as well [40–43]. There are, however, a number important limitations. For proper interpretation, oxygen consumption should be considered, which varies in the perioperative period to a great extent [40]. In addition, S_cv_O_2_ is usually intermittently determined, since special oximetry probes are needed for continuous S_cv_O_2_ measurement. Therefore, S_cv_O_2_ was not chosen as target in the treatment algorithm, but may be used additionally according to local practice.

The EGDT algorithm starts in the OR after induction of anesthesia before the start of surgery. Postoperatively in the ICU/PACU, EGDT continues until discharge for a maximum of 24 hours. In most hospitals, it is common practice to monitor patients after high-risk surgery for this period. For practical reasons therefore, EGDT can easily be implemented. More importantly, impaired tissue oxygenation may present up to 24 hours after surgery [44–46].

#### Standard care

We decided to limit the criteria for standard care in order to keep care in the control group close to local routine. As described above, EGDT is meant as a supplement to standard care, not as a substitute. For reasons of generalizability to other institutions, care in the control group should not deviate from local routine to a great extent. On the other hand, bias or major differences between the participating centers may occur if standard care is rather heterogeneous. Criteria for mechanical ventilation were introduced to prevent bias from differences between the groups, since a reliable SVV measurement requires specific ventilation settings [14, 15]. The other criteria are very routine in most institutions. The use of epidural analgesia, enhanced recovery after surgery (ERAS) protocols, corrections for preoperative fasting, and basic fluid regimes are not obligatory. Due to randomization, these treatment elements are expected to be balanced between the control and intervention groups. In addition, these elements do not interfere with the application of EGDT.

It would be valuable to monitor CO in the control group as well, albeit blinded for the clinicians. The presence or absence of differences in outcome between the control and intervention group are easier to interpret in the light of differences in CO between the groups. In clinical practice however, it may be difficult to apply standard care in the control group in the presence of a CO monitor. We fear that clinicians may tend to take CO values into consideration in their therapy. In addition, CO monitoring is associated with significant costs, which increases the total costs of the trial to a great extent.

#### Safety

Our systematic review of the literature did not reveal any harm due to the use of fluid or inotropes in goal-directed strategies. In the OR and ICU/PACU, both fluid and inotropes are extensively used. We decided to allow the use of both crystalloid and colloid fluids, according to local preferences. Norepinephrine and dobutamine were chosen for inotropic support as these inotropes are commonly used. Clinicians and other healthcare personnel are therefore familiar with the administration, clinical effects, and adverse reactions. In the algorithm however, patients may end up in a continuing loop of fluid or inotropic support if the response in CI is insufficient. In this case, volume overloading or adverse effects of inotropes may occur, and a number of safety measures have been incorporated in the algorithm in order to address this. If tachycardia, arrhythmia, myocardial ischemia, pulmonary edema, or cardiac decompensation is suspected or observed, any intervention within the algorithm should be stopped immediately. In contrast, fluid or inotropes should not be withheld in patients with CI values above their target. We therefore emphasize that EGDT is added to standard care and does not replace it. Interventions within the usual care for high-risk surgical patients, such as treatment of hypotension, hypovolemia, or any other suspicion of hemodynamic deterioration, should therefore be applied irrespective of the CI or the treatment algorithm.

### Methodological aspects

#### RCT design

Especially in studies evaluating perioperative hemodynamic therapy, randomized controlled trials (RCTs) have a number of limitations, such as impossibility to blind at the patient and caregiver level, and the Hawthorne effect [34, 47, 48]. The Hawthorne effect is a phenomenon whereby clinicians tend to improve care for patients included in studies, which may affect outcome in both the control and intervention groups [49]. We discussed the alternative approach of a before-after study. For the purposes of our study, prospective data collection is necessary. Therefore, the Hawthorne effect will be present in both ‘before’ and ‘after’ periods. More importantly, postoperative outcome after high-risk surgery is multifactorial. It is simply impossible to keep all these factors constant during the entire study period, which was the main reason for choosing the RCT design. Since the start of our trial, a number of important aspects in the care for high-risk surgical patients have changed. First, robotic surgery is increasingly being used in our institutions. Second, surgical safety checklists have been introduced in many centers, reducing both mortality and morbidity after surgery [50]. In one of the participating centers in this trial this led to a significant reduction in 30-day in-hospital mortality [51]. Similar checklists have been implemented in the other participating centers. We therefore regarded randomization to be crucial in this study. We stratified the randomization procedure with respect to participating center and type of surgery, since these covariates may influence outcome to a great extent. The number of patients in certain strata may become small however, which was the reason for applying blocked randomization.

#### Primary endpoint

The primary outcome measure in this trial is a combined endpoint of major complications in the first 30 days after surgery, including mortality. The use of composite endpoints in trials is common in order to increase statistical power and to capture the net benefit of the intervention [52, 53]. The most important disadvantage of this approach is that interpretation of the results is difficult if the outcomes are of different importance to patients. The components of a composite endpoint should therefore be clearly defined, clinically important, effected by the intervention, as homogeneous as possible, and weighed to reflect their relative importance [53].

The major complications embedded in the composite endpoint in our study are clearly defined and associated with increased long-term morbidity and mortality. Yet there are differences in terms of clinical consequences for the patient. Therefore, we decided to take the number of complications per patient as a primary endpoint, instead of the number of patients with one or more complications. In this way, the severity of the complication is reflected. For instance, if a patient develops pneumonia, which is treated with antibiotics in the ward, there is only one complication registered. If the patient however needs ICU readmission because of respiratory failure and septic shock, three complications are registered. We will report all complications separately to show if a difference in the primary endpoint is predominantly caused by one or more specific complications. In addition, the complications will be weighed using the Accordion complication severity score [31].

A number of other outcome measures have been considered as a primary endpoint in this trial. First, we performed a sample size calculation for the detection of a difference in mortality. This however revealed that over 5,000 patients would be needed, which was not feasible. Second, we calculated that approximately 250 patients would be needed to detect a difference in length of stay in the hospital. As a result, the study would have insufficient power to detect differences in terms of morbidity, which was our primary aim. Third, a single complication taken as an outcome measure was considered. In this case, however, consequences for the patient may differ considerably as well. As described previously, pneumonia may have a mild clinical course, but may also result in septic shock with multi-organ failure. Finally, we considered complications weighed according to the Accordian complication severity score as a primary outcome measure [31]. This, however, requires an ordinal approach to data-analysis, which is not straightforward. In addition, it remains difficult to compare patients with multiple complications.

#### Blinding

Ideally, blinding is used at the level of the patient, the caregiver, and the assessor of the outcome variable. In our trial however, blinding at the patient and caregiver is simply impossible. In theory, it is possible to use blinded assessors to determine outcome measures according to specified criteria. However, patient follow-up should be active and thorough, in order not to overlook complications and adverse outcomes once the patient is in the ward or at home. This requires frequent visits to the patients and monitoring of the patients’ medical records. During these visits and in these records, information about the use of CO monitoring and PLR testing is easily obtained, which makes blinding impossible. Therefore, all variables are elaborately defined, especially outcome parameters. Strict criteria are used to define the complications embedded in the composite primary endpoint. In this way, we aim to minimize bias as much as possible.

#### Discharge criteria

The length of stay in the hospital and ICU/PACU are important secondary endpoints, since they reflect the clinical course in the postoperative period and represent a significant part of the healthcare costs per patient. The discharge from patients from the hospital or ICU/PACU is, however, not only based on medical conditions but also influenced by the availability of care at the discharge destination. Continuation of care after treatment in the ICU/PACU depends on the capacity of the wards or intermediate care units. Discharge from the hospital may require adjustments at home or (temporary) placement in nursing homes. In addition, discharge from the IC/PACU occurs mainly the morning after surgery, which leads to a skewed distribution. To account for these effects, fit-for-discharge criteria from the hospital and ICU/PACU were determined (Additional file 3) [5].

## Trial status

Patient recruitment started in May 2012 and is expected to end in 2016. At the end of 2013, approximately 160 patients had been recruited across three centers. The fourth center is expected to start patient recruitment in 2014.

## Electronic supplementary material

Additional file 1: **Systematic review of the literature, performed before the start of the study.** (DOCX 82 KB)

Additional file 2: **The ‘4-5-6’ rule for transfusion with erythrocytes during acute, normovolemic anemia.** (DOCX 13 KB)

Additional file 3: **Fit-for-discharge criteria ICU/PACU.** (DOCX 14 KB)

Additional file 4: **IRB approval information.** (DOCX 14 KB)

## Abbreviations

ASA: American Society of Anesthesiologists
AWA: Arterial waveform analysis
CI: Cardiac index
CO: Cardiac output
CT: Computed tomography
DSMB: Data safety monitoring board
EGDT: Early goal-directed therapy
FC: Fluid challenge
GCP: Good clinical practice
HR: Heart rate
IBW: Ideal body weight
ICER: Incremental cost-effectiveness ratio
ICU: Intensive care unit
ICUR: Incremental cost-utility ratio
IRB: Institutional review board
MAP: Mean arterial pressure
OR: Operating room
QALY: Quality-adjusted life years
QOL: Quality of life
PACU: Post anesthesia care unit
PLR: Passive leg raising
SAE: Serious adverse event
SOFA: Sequential organ failure assessment
SVV: Stroke volume variation
TEE: Transesophageal echocardiography
TISS: Therapeutic intervention scoring system.

## References

[1] Pearse RM, Harrison DA, James P, Watson D, Hinds C, Rhodes A, Grounds RM, Bennett ED (2006). Identification of the high-risk surgical population in the United Kingdom. Crit Care.

[2] Jhanji S, Thomas B, Ely A, Watson D, Hinds CJ, Pearse RM (2008). Mortality and utilisation of critical care resources amongst high-risk surgical patients in a large NHS trust. Anaesthesia.

[3] Khuri SF, Henderson WG, DePalma RG, Mosca C, Healey NA, Kumbhani DJ (2005). Determinants of long-term survival after major surgery and the adverse effect of postoperative complications. Ann Surg.

[4] Benes J, Chytra I, Altmann P, Hluchy M, Kasal E, Svitak R, Pradl R, Stepan M (2010). Intraoperative fluid optimization using stroke volume variation in high risk surgical patients: results of prospective randomized study. Crit Care.

[5] Scheeren TW, Wiesenack C, Gerlach H, Marx G (2013). Goal-directed intraoperative fluid therapy guided by stroke volume and its variation in high-risk surgical patients: a prospective, randomized multicentre study. J Clin Monit Comput.

[6] Pearse R, Dawson D, Fawcett J, Rhodes A, Grounds RM, Bennett ED (2005). Early goal-directed therapy after major surgery reduces complications and duration of hospital stay. A randomized, controlled trial. Crit Care.

[7] Older P, Hall A, Hader R (1999). Cardiopulmonary exercise testing as a screening test for perioperative management of major surgery in the elderly. Chest.

[8] Kusano C, Baba M, Takao S, Sane S, Shimada M, Shirao K, Natsugoe S, Fukumoto T, Aikou T (1997). Oxygen delivery as a factor in the development of fatal postoperative complications after oesophagectomy. Br J Surg.

[9] Shoemaker WC (1972). Cardiorespiratory patterns of surviving and nonsurviving postoperative patients. Surg Gynecol Obstet.

[10] Peerless JR, Alexander JJ, Pinchak AC, Piotrowski JJ, Malangoni MA (1998). Oxygen delivery is an important predictor of outcome in patients with ruptured abdominal aortic aneurysms. Ann Surg.

[11] De Waal EE, De Rossi L, Buhre W (2006). Pulmonary artery catheter in anaesthesiology and intensive care medicine. Anaesthesist.

[12] Connors AF, Speroff T, Dawson NV, Thomas C, Harrell FE, Wagner D, Desbiens N, Goldman L, Wu AW, Califf RM, Fulkerson WJ, Vidaillet H, Broste S, Bellamy P, Lynn J, Knauw WA (1996). The effectiveness of right heart catheterization in the initial care of critically ill patients. SUPPORT Investigators JAMA.

[13] De Waal EE, Wappler F, Buhre WF (2009). Cardiac output monitoring. Curr Opin Anaesthesiol.

[14] Montenij LJ, de Waal EEC, Buhre WF (2011). Arterial waveform analysis in anesthesia and critical care. Curr Opin Anaesthesiol.

[15] Michard F, Teboul JL (2000). Using heart-lung interactions to assess fluid responsiveness during mechanical ventilation. Crit Care.

[16] Marik PE, Cavalazzi R, Vasu T, Hirani A (2009). Dynamic changes in arterial waveform derived variables and fluid responsiveness in mechanically ventilated patients: a systematic review of the literature. Crit Care Med.

[17] Jhanji S, Pearse RM (2009). The use of early intervention to prevent postoperative complications. Curr Opin Crit Care.

[18] Hamilton MA, Cecconi M, Rhodes A (2011). A systematic review on the use of preemptive hemodynamic intervention to improve postoperative outcomes in moderate and high-risk surgical patients. Anesth Analg.

[19] Cannesson M, Pestel G, Ricks C, Hoeft A, Perel A (2011). Hemodynamic monitoring and management in patients undergoing high risk surgery: a survey among North American and European anesthesiologists. Crit Care.

[20] Richard C, Monnet X, Teboul JL (2011). Pulmonary artery catheter monitoring in 2011. Curr Opin Crit Care.

[21] Bartha E, Davidson T, Hommel A, Thorngren KG, Carlsson P, Kalman S (2012). Cost-effectiveness analysis of goal-directed hemodynamic treatment of elderly hip fracture patients. Anesthesiology.

[22] Ebm C, Cecconi M, Sutton L, Rhodes A (2014). A cost-effectiveness analysis for postoperative goal-directed therapy in high-risk surgical patients. Crit Care Med.

[23] Salzwedel C, Puig J, Carstens A, Bein B, Molnar Z, Kiss K, Hussain A, Belda J, Kirov MY, Sakka SG, Reuter DA (2013). Perioperative goal-directed hemodynamic therapy based on radial arterial pulse pressure variation and continuous cardiac index trending reduces postoperative complications after major abdominal surgery: a multi-center, prospective, randomized study. Crit Care.

[24] Monnet X, Teboul JL (2008). Passive leg raising. Intensive Care Med.

[25] Tabot J, Teboul JL, Richard C, Monnet X (2009). Passive leg raising for the prediction of fluid responsiveness: importance of the postural change. Intensive Care Med.

[26] Monnet X, Rienzo M, Osman D, Anguel N, Richard C, Pinsky MR, Teboul JL (2006). Passive leg raising predicts fluid responsiveness in the critically ill. Crit Care Med.

[27] Thygesen K, Alpert JS, Jaffe AS, Simoons ML, Chaitman BR, White HD (2012). Joint ESC/ACCF/AHA/WHF Task Force for the Universal definition of myocardial infarction. Eur Heart J.

[28] New PW, Buchbinder R (2006). Critical appraisal and review of the Rankin scale and its derivatives. Neuroepidemiology.

[29] Mehta RL, Kellum JA, Shah SV, Molitoris BA, Ronco C, Warnock DG, Levin A (2007). Acute Kidney Injury Network: report of an initiative to improve outcomes in acute kidney injury. Crit Care.

[30] Levy MM, Fink MP, Marshall JC, Abraham E, Angus D, Cook D, Cohen J, Opal SM, Vincent JL, Ramsay G (2003). SCCM/ESICM/ACCP/ATS/SIS International Sepsis Definitions Conference. Crit Care Med.

[31] Strasberg SM, Linehan DC, Hawkins WG (2009). The Accordion severity grading system of surgical complications. Ann Surg.

[32] Ferreira FL, Bota DP, Bross A, Mélot C, Vincent JL (2001). Serial evaluation of the SOFA score to predict outcome in critically ill patients. JAMA.

[33] Keene AR, Cullen DJ (1983). Therapeutic Intervention Scoring System: Update 1983. Crit Care Med.

[34] MacDonald N, Pearse RM (2011). Peri-operative hemodynamic therapy: only large clinical trials can resolve our uncertainty. Crit Care.

[35] Kirov MY, Kuzkov VV, Molnar Z (2010). Perioperative haemodynamic therapy. Curr Opin Crit Care.

[36] Bendjelid K, Giraud R, Siegenhalter N, Michard F (2010). Validation of a new transpulmonary thermodilution system to assess global end-diastolic volume and extravascular lung water. Crit Care.

[37] Broch O, Renner J, Hocker J, Gruenewald M, Meybohm P, Schöttler J, Steinfath M, Bein B (2011). Uncalibrated pulse power analysis fails to reliably measure cardiac output in patients undergoing coronary artery bypass grafting. Crit Care.

[38] Brandfonbrener M, Landowne M, Shock NW (1955). Changes in cardiac output with age. Circulation.

[39] Boss GR, Seegmiller E (1981). Age-related physiological changes and their clinical significance. West J Med.

[40] Van Beest P, Wietasch G, Scheeren T, Spronk P, Kuiper M (2011). Clinical review: use of venous oxygen saturations as a goal: a yet unfinished puzzle. Crit Care.

[41] Shah S, Ouellette DR (2010). Early goal-directed therapy for sepsis in patients with preexisting left ventricular dysfunction: a retrospective comparison of outcomes based upon protocol adherence. Chest.

[42] Rivers E, Nguyen B, Havstad S, Ressler J, Muzzin A, Knoblich B, Peterson E, Tomlanovich M (2001). Early goal-directed therapy in the treatment of severe sepsis and septic shock. N Engl J Med.

[43] Donati A, Loggi S, Preiser JC, Orsetti G, Münch C, Gabbanelli V, Pelaia P, Pietropaoli P (2007). Goal-directed intraoperative therapy reduces morbidity and length of hospital stay in high-risk surgical patients. Chest.

[44] Lobo SM, Salgado PF, Castillo VG, Borim AA, Polachini CA, Palchetti JC, Brienzi SL, De Oliveira GG (2000). Effects of maximizing oxygen delivery on morbidity and mortality in high-risk surgical patients. Crit Care Med.

[45] Boyd O, Grounds RM, Bennett ED (1993). A randomized clinical trial of the effect of deliberate perioperative increase of oxygen delivery on mortality in high-risk surgical patients. JAMA.

[46] Shoemaker WC, Appel PL, Kram HB, Waxman K, Lee TS (1988). Prospective trial of supranormal values of survivors as therapeutic goals in high-risk surgical patients. Chest.

[47] Berguer R (2004). The evidence thing. Ann Vasc Surg.

[48] Michard F, Cannesson M, Vallet B (2011). Perioperative hemodynamic therapy: quality improvement programs should help resolve our uncertainty. Crit Care.

[49] Izawa MR, French MD, Hedge A (2011). Shining new light on the Hawthorne illumination experiments. Hum Factors.

[50] De Vries EN, Prins HA, Crolla RM, De Outer AJ, Van Andel G, Van Helden SH, Schlack WS, Van Putten MA, Gouma DJ, Dijkgraaf MG, Smorenburg SM, Boermeest MA (2010). Effect of a comprehensive surgical safety system on patient outcomes. N Engl J Med.

[51] Van Klei WA, Hoff RG, Van Aarnhem EE, Simmermacher RK, Regli LP, Kappen TH, Van Wolfswinkel L, Kalkman CJ, Buhre WF, Peelen LM (2012). Effects of the introduction of the WHO “Surgical Safety Checklist” on in-hospital mortality: a cohort study. Ann Surg.

[52] Freemantle N, Calvert M, Wood J, Eastaugh J, Griffin C (2003). Composite outcomes in randomized trials: greater precision but with greater uncertainty?. JAMA.

[53] Ferreira-González I, Permanyer-Miralda G, Busse JW, Bryant DM, Montori V, Alonso-Coello P, Walter SD, Guyatt GH (2007). Methodologic discussions for using and interpreting composite endpoint are limited, but still identify major concerns. J Clin Epidemiol.

