# Supplementary material for: Influence of early goal-directed therapy using arterial waveform analysis on major complications after high-risk abdominal surgery: study protocol for a multicenter randomized controlled superiority trial
Source: Trials. 2014 Sep 16;15:360. doi: 10.1186/1745-6215-15-360 (PMC4175278; doi:10.1186/1745-6215-15-360)
Supplement: Supplementary file 1 — Additional file 1: Systematic review of the literature, performed before the start of the study. (DOCX 82 KB) [file 13063_2014_2230_MOESM1_ESM.docx]

**APPENDIX A**

**Early goal-directed therapy using arterial waveform analysis in high-risk, abdominal surgery: a systematic review of the literature.**

**Search**

A systematic review of the literature was performed to evaluate all available evidence on the use of EGDT in high-risk, abdominal surgery. MEDLINE (provider: PUBMED) was searched using the keywords “goal-directed therapy AND surgery”, “high-risk surgery AND hemodynamic monitoring”, “high-risk surgery AND oxygen delivery”, “high-risk surgery AND cardiac output”, and “high-risk surgery AND stroke volume”. We limited our search to randomized controlled trials in the English language, published between 1985 and 2011. The title and abstracts identified were then screened for potential articles. After this primary exclusion, full articles were obtained and assessed for eligibility. In addition, the reference lists of identified trials were screened and another expert in the field was consulted to complete the list of publications.

**Selection criteria**

*Patient criteria*

Patients undergoing elective, high-risk, abdominal surgery were eligible. Other types of surgery (e.g. cardiac, brain, peripheral vascular or orthopedic surgery) were excluded. In addition, studies in patients suffering from sepsis, trauma, or other critically illnesses not undergoing surgery were beyond the scope of this review.

*Intervention criteria*

The intervention comprised of measurement and subsequent maintenance of CO or a CO-derived variable above a clearly defined, preset, target value. CO-derived variables included cardiac index (CI), stroke volume (SV) or stroke volume index (SVI), and tissue oxygen delivery (DO_2_) or tissue oxygen delivery index (DO_2_I). Studies comparing two or more fluid regimes, inotropic support, or other interventions without the use of a target variable were excluded. Therapy in the control group needed to be standard care with or without complementary requisites on fluid or inotropic support. Studies using another goal-directed strategy in the control group were excluded. No restrictions on the duration and timing of the intervention (before, during or after the operation, or any combination) were imposed. As a primary endpoint, patient outcome (mortality, morbidity), hospital data (length of stay in the hospital) and hemodynamic variables were accepted.

*Assessment*

Eligible studies were graded using the systems described by Jadad et al [I]. This scale is used to assess study quality by scoring three elements (randomization, double blinding, and withdrawals and dropouts) with a score range of 1 to 5. With respect to outcome measures, the effects of goal-directed strategies on mortality, morbidity, and length of stay in the hospital was evaluated. In addition, the studies were reviewed for adverse effects, use of inotropic support and fluids, and hemodynamic parameters. Care in the control and intervention group was evaluated with respect to the use of central venous pressure (CVP) and pulmonary capillary wedge pressure (PCWP) to determine cardiac preload, and with respect to user-friendliness.

**Results**

*Search and selection*

Our search strategy retrieved 2799 titles suitable for primary review (figure I). On the basis of titles and abstract, 2.768 studies in children or animals, and studies concerning goal-directed therapy strategies within the field of sepsis, trauma without surgery, and cardiac surgery were excluded. The remaining 31 studies were assessed in full text. Screening the reference lists of these studies did not lead to additional selection of articles. Of the 31 studies assessed, 22 did not meet the eligibility criteria and were excluded: 1 study investigating emergency (non-elective) surgery, 5 studies concerning another type of surgery than abdominal, 11 studies not using CO or a CO-derived variable as target, and 5 studies comparing different therapy strategies without the use of a preset hemodynamic target variable. The number of studies obtained for qualitative analysis was therefore 9 (table I) [II-X]. The final list of studies was presented to the three experts and checked for completeness, which did not lead to the inclusion of additional studies.

*Quality assessment*

The final list included 1 grade I, 2 grade II, 5 grade III, and 1 grade IV. The main limitations reducing the quality scores were inability for full blinding and insufficient reporting of withdrawals and dropouts. Especially blinding of the awake patient in the post-operative period was often considered not feasible. In general, the randomization procedure was adequately performed and well documented.

*Outcome measures*

The primary outcome measures reported were mortality, the incidence of complications and length of stay in the hospital (LOS). All studies reported mortality as an endpoint. A significant reduction in mortality was reported in 4 studies [VI,VII,IX,X]. Mortality in the control group varied between 17% and 50%, in comparison with 3% and 21% in the intervention group. The number of complications per patient was significantly reduced in 4 studies, with the number of complications per patient varying between 1.03 and 1.5 in the control group, and 0.39 and 0.97 in the intervention group [II,III,IX,X]. In addition, the number of patients developing one or more complications was reduced in 4 studies [II,III,VI,VII]. There were 2 studies reporting a significant reduction in LOS [III,VII]. One study did not show any benefit in outcome (mortality, morbidity, or LOS) [IV]. None of the studies included a quality of life assessment and cost-effectiveness analysis or long-term follow-up.

*Adverse events*

Five studies reported adverse events in the intervention group [III-V,VIII,X]. A significant increase in pulmonary embolism was seen (8 vs. 0, p= 0.004) in one study, most likely due to the use of PACs [IV]. Two additional studies reported transient arrhythmias during PAC insertion [V,X]. Other reported complications associated with PAC use were infection at the catheter insertion site, pneumothorax, and sepsis [VIII,X]. The study by Valentine et al. reported a significant increase in the number of patients with complications in the intervention group [VIII]. These complications included prolonged hypotension, new arrhythmias, sustained tachycardia, sustained bradycardia, and bronchospasm. One study reported myocardial ischemia due to use of dopexamine, which disappeared after stopping the infusion [III]. In the other studies however, the results point to a lower incidence of myocardial ischemia, although significant reductions have not been reported.

*Goal-directed treatment and its effects*

Four studies reported a significant increase in tissue oxygen delivery and/or CO in the intervention group [III,VI,IX,X]. In the other studies, CO and hence tissue oxygen delivery was not measured in the control group [IV,V,VII,VIII], or differences were not seen [II]. Goal-directed treatment resulted in differences in the use of inotropes and fluids in a majority of studies [II,III-VI,VIII,IX]. CVP and/or PCWP were used as a hemodynamic target in the control and/or intervention group in many studies. The most recent study uses a rather extensive treatment algorithm, taking multiple hemodynamic parameters and targets into account [II].

**Interpretation**

These results indicate that EGDT is a promising strategy to reduce morbidity and even mortality in high-risk surgery. There are however several limitations which may explain why EGDT has never been widely implemented into routine practice.

First, the results from most studies are outdated, and not applicable to the current standard of care. Studies reporting a significant reduction in mortality were performed in the period 1988 to 2000. Mortality in the control group was around 30%, which does not correspond to mortality rates in high-risk surgery nowadays (around 5%). Even the reduced mortality rate in the intervention group (around 11%) in those studies is widely above current mortality. A similar effect is seen in studies reporting a reduction in the number of complications per patient. The mean number of complications in the control group (around 1.1) is above current complications rates (around 0.5 per patient). The length of stay in the hospital (LOS) varies considerably with respect to hospital type and the type of surgery. It is however assumed that the current LOS for high-risk surgical patients is beneath the numbers reported in most of the studies (LOS in the control group around 15 days).

Second, invasive and difficult instruments have been used to measure the EGDT target variable. In 7 studies, CO or the CO-derived target parameter is measured using a pulmonary artery catheter (PAC) To measure CO, the thermodilution technique with a pulmonary artery catheter (PAC) remains one of the most reliable methods, but its use is associated with severe complications (cardiac arrhythmias, pulmonary bleeding and infection), and definite evidence on its efficacy in high-risk, non-cardiac surgery is lacking [XI]. Therefore, the use of the PAC is restricted to specific types of extended cardiac surgery, and discouraged to be used in other types of surgery [XI,XII]. As a result, clinicians are reserved with the application of PACs in daily practice. In this systematic review, complications due to PAC use were reported in 4 studies [IV,V,VIII,X]. [IV,V,VIII,X

Third, CVP and PCWP were used as a target parameter in many studies. These static preload indices have been shown to be unreliable in predicting fluid responsiveness [XIII]. Consequently, CVP and PCWP should not be used in goal-directed treatment algorithms or in the control group.

Only 2 studies are available which use the minimally invasive and easy-to-use arterial waveform technique to measure CO for EGDT [II,III]. These studies demonstrate significant reductions in LOS and the incidence of complications. However, minor complications such as short lasting intra-operative hypotension or urinary tract infection are included as outcome measure. These complications are not associated with increased long-term morbidity and mortality, and should be evaluated separately. In addition, the number of included patients was small (in between 60 and 122), and the studies were performed in one center. Therefore, the generalizability of the results to other institutions is limited.

One final but major limitation applies to all of the studies described. They lack information about the effect of EGDT on long-term outcome, quality of life and utilization of healthcare resources, which hinders a cost-effectiveness analysis.

**Conclusion**

This systematic search of the literature confirms that EGDT is promising to improve outcome after high-risk surgery. The results and methods of most prior studies are however not maintainable or applicable in the current or future patient facing a high-risk surgical intervention. Arterial waveform analysis has become a respectable alternative to the older invasive and impractical techniques, and is promising to serve as a base for EGDT. Definitive evidence on its efficacy and cost-effectiveness is lacking, which points to the urgent need for an elaborate study on the abilities of arterial waveform analysis to improve outcome after high-risk surgery.

*Figure I. The results from the systematic search, before and after exclusion based on title and abstract, and after fulltext assessment.*

**
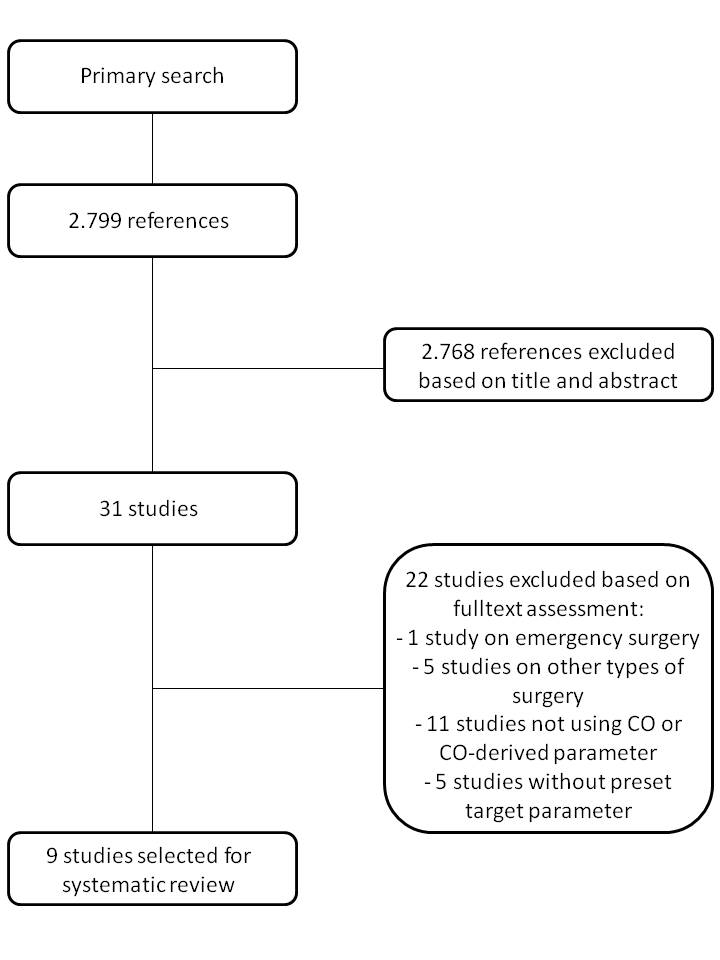
**

*Table I.A. Overview of the studies included in the systematic review, with respect to quality score, patients, intervention, and consequences of goal-directed treatment in terms of hemodynamic variables and therapy.*

*Abbreviations: CI = cardiac index, SVV = stroke volume variation, DO_2_ = oxygen delivery, DO_2_I = oxygen delivery index, AWA = arterial waveform analysis, PAC= pulmonary artery catheter, MAP = mean arterial pressure*

| **Reference** | **Quality score** | **Patients** | | **Intervention** | | **Main results (intervention compared to control)** | |
| --- | --- | --- | --- | --- | --- | --- | --- |
|  |  | *number* | *high-risk procedure, patient, or both* | *target variable* | *monitoring device* | *hemodynamic variables* | *fluid and inotropic support* |
| Benes (2010) | 3 | 120 | both | CI, SVV | AWA | no differences | more colloid intraoperatively |
| Pearse (2005) | 3 | 122 | both | DO_2_ | AWA | DO_2_ and SV higher | more colloid and dopexamine |
| Sandham (2003) | 3 | 1994 | both | DO_2_I | PAC | not provided | more inotropes |
| Bonazzi (2002) | 2 | 100 | procedure | CI, DO_2_ | PAC | not provided | more fluid |
| Lobo (2000) | 3 | 37 | both | DO_2_ | PAC | CI and DO_2_ higher | more dobutamine, less dopamine |
| Wilson (1999) | 4 | 138 | both | DO_2_ | PAC | not provided | no difference |
| Valentine (1998) | 3 | 120 | procedure | CI | PAC | not provided | more fluid |
| Boyd (1993) | 1 | 107 | both | DO_2_ | PAC | CI and DO_2_ higher preoperatively | more fluid preoperatively |
| Shoemaker (1988) | 2 | 398 | both | CI, DO_2_ | PAC | CI and DO_2_ higher | not provided |

*Table I.B. Overview of the studies included in the systematic review, with respect to outcome measures and adverse effects. (*) refers to the primary endpoint*

*Abbreviations: LOS = length of stay in the hospital, N.S. = not significant, PVC = premature ventricular complex, pt(s) = patient(s)*

| **Reference** | **Main results (intervention vs control)** | | | |
| --- | --- | --- | --- | --- |
|  | *mortality* | *complications* | *LOS* | *adverse effects* |
| Benes (2010) | 1.7 vs 3.3% (N.S.) | 30 vs 58.3% pts with complication(s) (p=0.003) (*); 0.57 vs 1.28 complications per pt (p=0.007) | 9 vs 10 days (N.S.) | no |
| Pearse (2005) | 28 days: 9.7 vs 11.7 (p=0.78) | 0.7 vs 1.5 complication per pt (p=0.002); 44 vs 68% pts with complication(s) (p=0.007) | hospital: 11 vs 14 days (p=0.001) (*) | 1 pt with myocardial ischemia |
| Sandham (2003) | in-hospital: 7.8 vs 7.7% (*) | no difference except pulmonary embolism (adverse effect) | 10 days both | 8 vs 0 pts with pulmonary embolism (p=0.004) |
| Bonazzi ^(1)^ (2002) | in-hospital: both 0% | 4 vs 8% (N.S.) | 12 vs 11 (N.S.) | 9 vs 0 pts with transient PVC’s during PAC insertion |
| Lobo (2000) | 28 days: 15.7 vs 33% (N.S.) (*); 60 days: 15.7 vs 50% (p<0.05) | 32 vs 67% pts with complication(s) (p<0.05) | not provided | no |
| Wilson ^(2)^ (1999) | in-hospital: 3 vs 17% (p=0.007) (*) (both groups vs control) | 30 vs 61% pts with complication(s) dopexamine group vs control (p<0.05) | reduced in both groups vs control (numbers not provided) | no |
| Valentine ^(3)^ (1998) | in-hospital: 5 vs 2% (N.S.) | 11 vs 3% pts with complication(s) (p=0.02) | 13 days both | 8 (13%) pts with PAC related complications (transient arrhythmia, pneumothorax, sepsis) |
| Boyd (1993) | 28 days: 5.7 vs 22.2% (p=0.015) (*) | 0.68 vs 1.35 complications per pt (p=0.008) | hospital: 12.5 vs 16 days (N.S.) | no |
| Shoemaker ^(4)^ (1988) | In-hospital: series 1: 21 vs 38% (p<0.05); series 2: 4 vs 33 (p<0.05) vs 23% (p>0.05) | series 1: 0.97 vs 1.54 (p<0.05); series 2:  0.39 (p<0.05) vs 1.3 vs 1.03 (p<0.05), complications per pt | no differences | 12% transient arrhythmias during PAC placement, 4% insertion site infection |

^(1)^ pilot study, no sample size calculation

^(2)^ two intervention groups: dopexamine and adrenaline

^(3)^ no sample size calculation performed

^(4)^ 2 patient series, series 1: PAC protocol vs PAC control; series 2: PAC protocol vs PAC control vs CVP control

**References**

1. Jadad AR, Moore RA, Carrol D, Jenkinson C, Reynolds DJ, Gavaghan DJ, McQuay HJ: **Assessing the quality of reports of randomized controlled trials: is blinding necessary?** *Control Clin Trials* 1996, **17**:1-12.
2. [Benes J](http://www.ncbi.nlm.nih.gov/pubmed?term=Benes%20J%5BAuthor%5D&cauthor=true&cauthor_uid=20553586), [Chytra I](http://www.ncbi.nlm.nih.gov/pubmed?term=Chytra%20I%5BAuthor%5D&cauthor=true&cauthor_uid=20553586), [Altmann P](http://www.ncbi.nlm.nih.gov/pubmed?term=Altmann%20P%5BAuthor%5D&cauthor=true&cauthor_uid=20553586), [Hluchy M](http://www.ncbi.nlm.nih.gov/pubmed?term=Hluchy%20M%5BAuthor%5D&cauthor=true&cauthor_uid=20553586), [Kasal E](http://www.ncbi.nlm.nih.gov/pubmed?term=Kasal%20E%5BAuthor%5D&cauthor=true&cauthor_uid=20553586), [Svitak R](http://www.ncbi.nlm.nih.gov/pubmed?term=Svitak%20R%5BAuthor%5D&cauthor=true&cauthor_uid=20553586), [Pradl R](http://www.ncbi.nlm.nih.gov/pubmed?term=Pradl%20R%5BAuthor%5D&cauthor=true&cauthor_uid=20553586), [Stepan M](http://www.ncbi.nlm.nih.gov/pubmed?term=Stepan%20M%5BAuthor%5D&cauthor=true&cauthor_uid=20553586): **Intraoperative fluid optimization using stroke volume variation in high risk surgical patients: results of prospective randomized study.** *Crit Care* 2010, **14**:R118.
3. Pearse R, Dawson D, Fawcett J, Rhodes A, Grounds RM, Bennett ED: **Early goal-directed therapy after major surgery reduces complications and duration of hospital stay. A randomized, controlled trial.** *Crit Care* 2005, **9**:687-693.
4. Sandham JD, Hull RD, Brant RF, Knox L, Pineo GF, Doig CJ, Laporta DP, Viner S, Passerini L, Devitt H, Kirby A, Jacka M: [**A randomized, controlled trial of the use of pulmonary-artery catheters in high-risk surgical patients.**](http://www.ncbi.nlm.nih.gov/pubmed/12510037) *N Engl J Med* 2003, **348**:5-14.
5. Bonazzi M, Gentile F, Biasi GM, Migliavacca S, Esposti D, Cipolla M, Marsicano M, Prampolini F, Ornaghi M, Sternjakob S, Tshomba Y: [**Impact of perioperative haemodynamic monitoring on cardiac morbidity after major vascular surgery in low risk patients. A randomised pilot trial.**](http://www.ncbi.nlm.nih.gov/pubmed/12027474) *Eur J Vasc Endovasc Surg* 2002, **23**:445-451.
6. Lobo SM, Salgado PF, Castillo VG, Borim AA, Polachini CA, Palchetti JC, Brienzi SL, De Oliveira GG: **Effects of maximizing oxygen delivery on morbidity and mortality in high-risk surgical patients.** *Crit Care Med* 2000, **28**:3396-3404.
7. Wilson J, Woods I, Fawcett J, Whall R, Dibb W, Morris C, McManus E: **Reducing the risk of major elective surgery: randomised controlled trial of preoperative optimisation of oxygen delivery.** *BMJ* 1999, **318**:1099-1103.
8. Valentine RJ, Duke ML, Inman MH, [Grayburn PA](http://www.ncbi.nlm.nih.gov/pubmed?term=Grayburn%20PA%5BAuthor%5D&cauthor=true&cauthor_uid=9510275), [Hagino RT](http://www.ncbi.nlm.nih.gov/pubmed?term=Hagino%20RT%5BAuthor%5D&cauthor=true&cauthor_uid=9510275), [Kakish HB](http://www.ncbi.nlm.nih.gov/pubmed?term=Kakish%20HB%5BAuthor%5D&cauthor=true&cauthor_uid=9510275), [Clagett GP](http://www.ncbi.nlm.nih.gov/pubmed?term=Clagett%20GP%5BAuthor%5D&cauthor=true&cauthor_uid=9510275): [**Effectiveness of pulmonary artery catheters in aortic surgery: a randomized trial.**](http://www.ncbi.nlm.nih.gov/pubmed/9510275) *J Vasc Surg* 1998, **27**:203-211.
9. Boyd O, Grounds RM, Bennett ED: [**A randomized clinical trial of the effect of deliberate perioperative increase of oxygen delivery on mortality in high-risk surgical patients.**](http://www.ncbi.nlm.nih.gov/pubmed/7907668) *JAMA* 1993, **270**:2699-2707.
10. Shoemaker WC, Appel PL, Kram HB, Waxman K, Lee TS; [**Prospective trial of supranormal values of survivors as therapeutic goals in high-risk surgical patients.**](http://www.ncbi.nlm.nih.gov/pubmed/3191758) *Chest* 1988, **94**:1176-1186.
11. De Waal EE, De Rossi L, Buhre W: **[Pulmonary artery catheter in anaesthesiology and intensive care medicine.]** *Anaesthesist* 2006, **31**:713-728.
12. Alhashemi JA, Cecconi M, Hofer CK: **Cardiac output monitoring: an integrated perspective.** *Crit Care* 2011, **15**:214-222.
13. Bendjelid K, Romand JA: **Fluid responsiveness in mechanically ventilated patients: a review of indices used in intensive care.** *Intensive Care Med* 2003, **29**:352-360.
