# Supplementary material for: Influence of early goal-directed therapy using arterial waveform analysis on major complications after high-risk abdominal surgery: study protocol for a multicenter randomized controlled superiority trial
Source: Trials. 2014 Sep 16;15:360. doi: 10.1186/1745-6215-15-360 (PMC4175278; doi:10.1186/1745-6215-15-360)
Supplement: Supplementary file 2 — Additional file 2: The ‘4-5-6’ rule for transfusion with erythrocytes during acute, normovolemic anemia. (DOCX 13 KB) [file 13063_2014_2230_MOESM2_ESM.docx]

**APPENDIX B**

**The “4-5-6” rule for transfusion with erythrocytes during acute, normovolemic anemia**

Consider tranfusion of the serum hemoglobine level is < 4 mmol⋅l^-1^ in the following circumstances:

- healthy patients (ASA physical status grade I)
- age < 60 years
- normovolemic blood loss in 1 location

Consider tranfusion of the serum hemoglobine level is < 5 mmol⋅l^-1^ in the following circumstances:

- healthy (ASA I) patients with normovolemic blood loss in 1 location, but with age > 60 years
- healthy (ASA I) patients with age < 60 years, but with normovolemic blood loss in more than 1 location
- patients with ASA physical health status grade II and III
- perioperative setting with expected blood loss > 500 ml
- patients with fever
- postoperatively after uncomplicated cardiac surgery

Consider tranfusion of the serum hemoglobine level is < 6 mmol⋅l^-1^ in the following circumstances:

- patients with ASA physical status grade IV
- patients with insufficient cardiopulmonary mechanisms to compensate hemodilution
- septic or toxic patients
- patients with severe pulmonary disease, or symptomatic cerebrovascular disease
