# Supplementary material for: Influence of early goal-directed therapy using arterial waveform analysis on major complications after high-risk abdominal surgery: study protocol for a multicenter randomized controlled superiority trial
Source: Trials. 2014 Sep 16;15:360. doi: 10.1186/1745-6215-15-360 (PMC4175278; doi:10.1186/1745-6215-15-360)
Supplement: Supplementary file 3 — Additional file 3: Fit-for-discharge criteria ICU/PACU. (DOCX 14 KB) [file 13063_2014_2230_MOESM3_ESM.docx]

**APPENDIX C**

**Fit-for-discharge criteria ICU/PACU**

1) Hemodynamically stable, without the need for inotropic support in high doses

2) Respiratory stable, as defined by:

- oxygen need with a fraction of inspired oxygen < 60%
- need for drainage or suction of saliva < 3 times per shift
- time after extubation: > 2 hours (extubation after regular period of postoperative mechanical ventilation) or > 24 hours (extubation after prolonged postoperative mechanical ventilation [> 24 hours])

3) neurologically: Glasgow Coma Score > 8 or stable neurological condition acceptable for receiving intermediate care unit or ward

4) other:

- stable production of surgical drains
- no need for continuous dialysis

**Fit-for-discharge criteria hospital**

1) Tolerance for oral feeding:

- at least one solid meal per day without nausea, vomiting or increase in abdominal pain
- active drinking without the need for intravenous fluid administration

2) Recovery of gastrointestinal function in terms of flatulence

3) Adequate analgesia with oral medication during resting, sitting and walking (if possible preoperatively) without significant pain (pain under controle according to the patient, or pain score ≤ 4 on the visual analog scale)

4) Ability for mobilization (sitting, standing, walking, and walking stairs if needed) and self-care (washing, visiting the toilet, getting dressed), unless this was impossible before surgery

5) No signs of complications or untreated medical problems:

- normal body temperature
- HR, blood pressure and breathing frequency comparable to preoperative values
- stable hemoglobine level
- normal bladder function, or comparable to preoperative function
