# Supplementary material for: Influence of early goal-directed therapy using arterial waveform analysis on major complications after high-risk abdominal surgery: study protocol for a multicenter randomized controlled superiority trial
Source: Trials. 2014 Sep 16;15:360. doi: 10.1186/1745-6215-15-360 (PMC4175278; doi:10.1186/1745-6215-15-360)
Supplement: Supplementary file 4 — Additional file 4: IRB approval information. (DOCX 14 KB) [file 13063_2014_2230_MOESM4_ESM.docx]

**APPENDIX D**

**IRB approval information**

The study has been approved by the Institutional Review Board of the University Medical Center Utrecht:

- protocol reference: 10/173
- date: March the 21^st^ 2011

Local approval has been obtained in the following participating centers:

- Albert Schweitzer Hospital, IRB of the Albert Schweitzer Hospital, reference Trial 2012.04 (EGDT), January the 24^th^
- University Medical Center Groningen, IRB of the University Medical Center Groningen, reference NL32416.041.10, January the 4^th^ 2012

Local IRB approval in the Maastricht University Medical Center is in preparation. Local approval will be obtained in any other additional participating center, before start of patient inclusion.
